# Evaluation of HBV-Like Circulation in Wild and Farm Animals from Brazil and Uruguay

**DOI:** 10.3390/ijerph16152679

**Published:** 2019-07-26

**Authors:** Yasmine R. Vieira, Moyra M. Portilho, Flávia F. Oliveira, Alexandro Guterres, Débora Regina L dos Santos, Lívia M. Villar, Santiago Mirazo, Juan Arbiza, Luana A.G. Dimache, Fernando Q. Almeida, Martha L. Brandão, José Luís P. Cordeiro, Fabiana L. Rocha, Fernanda C. Azevedo, Frederico G. Lemos, João Bosco V. Campos, Gabriel C. Macedo, Heitor M. Herrera, Igor Alexandre S. Péres, Namor P. Zimmermann, Ubiratan Piovezan, Aiesca O. Pellegrin, Vanessa S. de Paula, Marcelo A. Pinto

**Affiliations:** 1 Laboratório de Desenvolvimento Tecnológico em Virologia, Instituto Oswaldo Cruz (IOC), Fundação Oswaldo Cruz, Rio de Janeiro, RJ 21040-900, Brazil; 2 Laboratório de Hepatites Virais, Instituto Oswaldo Cruz (IOC), Fundação Oswaldo Cruz, Rio de Janeiro, RJ 21040-900, Brazil; 3 Laboratório de Virologia Molecular, Instituto Oswaldo Cruz (IOC), Fundação Oswaldo Cruz, Rio de Janeiro, RJ 21040-900, Brazil; 4 Laboratório de Hantaviroses e Rickettsioses, Instituto Oswaldo Cruz (IOC), Fundação Oswaldo Cruz, Rio de Janeiro, RJ 21040-900, Brazil; 5 Laboratório de Viroses Veterinárias, Departamento de Microbiologia Veterinária e Imunologia, Universidade Federal Rural do Rio de Janeiro (UFRRJ), Seropédica, RJ 23897-000, Brazil; 6 Sección Virología, Facultad de Ciencias, Universidad de la República, Montevideo 11400, Uruguay; 7 Departamento de Medicina e Cirurgia, Instituto de Veterinária, Universidade Federal Rural do Rio de Janeiro (UFRRJ), Seropédica, RJ 23897-000, Brazil; 8 Fiocruz Mata Atlântica, Fundação Oswaldo Cruz, Rio de Janeiro, RJ 22713-560, Brazil; 9 Centro de Ciências Aplicadas e Educação, Campus IV Litoral Norte, Universidade Federal da Paraíba, Rio Tinto, PB 58297-000, Brazil; 10 Programa de Conservação Mamíferos do Cerrado (PCMC), Cumari, GO 74690-900, Brazil; 11 Unidade Acadêmica Especial de Biotecnologia, Universidade Federal de Goiás, Goiânia, GO 74690-900, Brazil; 12 Laboratório de Biologia Parasitária, Universidade Católica Dom Bosco, Campo Grande, MS 79117-010, Brazil; 13 Embrapa Pantanal, CP 109, Corumbá, MS 79320-900, Brazil; 14 Faculdade de Medicina Veterinária e Zootecnia, Universidade Federal de Mato Grosso do Sul, Campo Grande, MS 79070-900, Brazil; 15 Embrapa Tabuleiros Costeiros, Aracaju, SE 49040-490, Brazil

**Keywords:** hepadnavirus, animal infectious diseases, disease impact, reverse zoonoses, real-time PCR

## Abstract

The origin of the hepatitis B virus is a subject of wide deliberation among researchers. As a result, increasing academic interest has focused on the spread of the virus in different animal species. However, the sources of viral infection for many of these animals are unknown since transmission may occur from animal to animal, human to human, animal to human, and human to animal. The aim of this study was to evaluate hepadnavirus circulation in wild and farm animals (including animals raised under wild or free conditions) from different sites in Brazil and Uruguay using serological and molecular tools. A total of 487 domestic wild and farm animals were screened for hepatitis B virus (HBV) serological markers and tested via quantitative and qualitative polymerase chain reaction (PCR) to detect viral DNA. We report evidence of HBsAg (surface antigen of HBV) and total anti-HBc (HBV core antigen) markers as well as low-copy hepadnavirus DNA among domestic and wild animals. According to our results, which were confirmed by partial genome sequencing, as the proximity between humans and animals increases, the potential for pathogen dispersal also increases. A wider knowledge and understanding of reverse zoonoses should be sought for an effective One Health response.

## 1. Introduction

The family *Hepadnaviridae* consists of hepatotropic enveloped viruses containing partially double-stranded circular DNA that replicates by reverse transcription [1]. These viruses are classified into the genera *Orthohepadnavirus* (mammals) and *Avihepadnavirus* (birds). Humans and non-human primates such as chimpanzees (*Pan troglodytes*), gibbons (*Hylobates* sp.), gorillas (*Gorilla gorilla*), orangutans (*Pongo pygmaeus*), and woolly monkeys (*Lagothrix lagotricha*) can be infected by hepatitis B virus (HBV), which is the prototype species [2,3,4,5,6,7]. HBV-like viruses are also found in a variety of other mammals, including woodchucks (WHV) [8], squirrels (GSHV/ASHV) [9,10], bats (RBHBV/HBHBV/TBHBV) [11], and birds such as ducks (DHBV), geese (GHBV), herons (HHBV), and storks (STHBV) [2,12,13,14,15,16,17,18].

Recent findings have confirmed the circulation of a virus similar to HBV in swine [19,20] and chickens [21] and demonstrated the presence of endogenous viral elements (EVEs) from hepadnaviruses in snakes [22,23], turtles, and crocodilians [22]. Furthermore, the creation of a novel genus assigned to an HBV-like virus circulating among fish has been proposed [24]. Based on these data, hepadnaviruses might be more ancient than previously thought (>200 million years) [25], and the list of candidate species that may serve as hepadnavirus hosts or reservoirs is longer than expected.

Human infection by HBV is endemic worldwide [26]. The World Health Organization (WHO) estimates that 240 million people are chronically infected with HBV [27], and 780,000 people die annually from the associated complications, such as cirrhosis and liver cancer [28]. Contact with blood or other body fluids from an infected person leads to the transmission of this pathogen [27]. Although immunization strategies have been adopted to prevent its spread [26], researchers question whether the existence of a virus shared by different host species impairs eradication attempts [29].

However, the sources of viral infection for many of these animals are unknown, and hepadnavirus donors and recipients have not been defined among humans and other animal species [30]. Although animal-to-human transmission is relatively well described, pathogenic traffic in the opposite direction is poorly understood. Reverse zoonosis or anthroponosis is a fascinating concept and a main global issue. Farm animals are transported far and wide and interact with wild species that they would never have encountered naturally. With a rapid growth in animal production and an increase in the movement of both animals and humans, a human pathogen could easily circulate and eventually adapt in different niches/hosts/species [31]. To provide insights about the range of HBV-like hosts, this study evaluated HBV-like circulation in a variety of wild and farm animals from different regions of Brazil and Uruguay.

## 2. Materials and Methods

### 2.1. Ethics Committee, Study Group, and Sample Collection

This study was approved by the Animal Use Ethics Committees (CEUA) of the institutions involved under the licenses CBA_02356_013, CEUA/UFRRJ n° 375/2013, CEUA/UFMS n° 500/2013, CEUA/UCDB n° 1/2013, and CEUA/UFG 086/2014. The capture of wild animals was licensed by the Instituto Chico Mendes de Conservação da Biodiversidade (SISBIO/ICMBio) under the licenses SISBIO/ICMBio 35296-2, SISBIO/ICMBio 49647-1, and SISBIO/ICMBio 14576-4) in accordance with Brazilian regulations. Appropriate biosecurity techniques and individual protective equipment were implemented during all procedures involving the collection and handling of biological samples.

**Figure 1:**
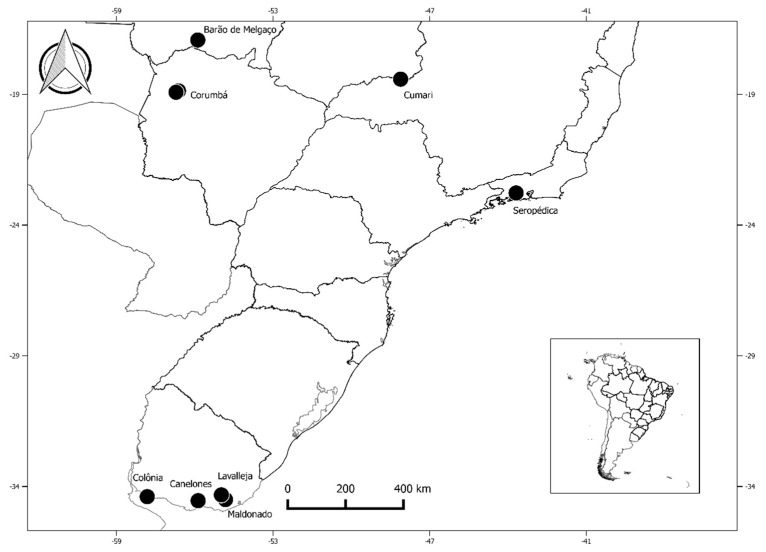
Collection sites of animals in the present study.

The study sample included 487 animals from different sites in Brazil and Uruguay that were divided into Groups A, B, and C according to the occurrence of human contact and the environment. Group A corresponded to 200 domestic animals from semi-extensive farms or confined conditions, and it included 75 domestic pigs (*Sus scrofa*) from the municipalities of Canelones, Lavalleja, and Colonia/Uruguay, and 125 equines (*Equus ferus caballus*) from the municipality of Seropédica/Brazil. Group B corresponded to 189 domestic animals that were raised in the wild or under free conditions, and it corresponded to 140 domestic dogs (*Canis lupus familiaris*) that are used for cattle work and hunting and have frequent access to farming and wild areas, and they were obtained from the municipalities of Corumbá/Brazil (Pantanal Biome) and Cumari/Brazil (Cerrado Biome). Finally, Group C corresponded to 98 free-roaming exotic and wild mammals, including 11 wild boars (*Sus scrofa*) from the municipality of Maldonado/Uruguay, 61 wild pigs (*Sus scrofa*) from the municipalities of Barão de Melgaço and Corumbá/Brazil (Pantanal Biome), one jaguar (*Panthera onca*) from the municipality of Barão de Melgaço/Brazil (Pantanal Biome), and 19 crab-eating foxes (*Cerdocyon thous*), four maned wolves (*Chrysocyon brachyurus*), one crab-eating raccoon (*Procyon cancrivorus*), and one hoary fox (*Lycalopex vetulus*) from the municipality of Cumari/Brazil (Cerrado Biome) (Figure 1).

Domestic animals were physically restrained. Wild species were actively captured using a tranquilizer gun or with Tomahawk box traps that were baited with sardines and boiled chicken, followed by chemical immobilization with an intramuscular injection of a combination of zolazepam and tiletamine (ZoletilH^®^, Virbac, São Paulo, Brazil) at dosages of 3–10 mg/kg depending on the target species. A total of 5 to 20 mL of venous blood was collected from the cephalic vein using Vacutainer^®^ (BD LifeSciences, Franklin Lakes, NJ, USA) 18–21 G needles and directly transferred into vacuum tubes containing a gel separator and clot activator (BD Vacutainer^®^ SST II Advance, BD LifeSciences, Franklin Lakes, NJ, USA). The tubes were refrigerated (4 °C) until they were processed. After 10–15 min of clot retraction, the samples were centrifuged at 3500 rpm for 5 min at room temperature (20–22 °C). The sera were separated and stored in aliquots at −20 and −80 °C until further analysis.

### 2.2. Evaluation of Serological Markers

The serum samples were screened using specific enzyme-linked immunosorbent assay (ELISA) for HBV serological markers (HBsAg, surface antigen of HBV; and total anti-HBc, an HBV core antigen) [32]. The immunoassay approaches included antigen capture for the HBsAg test and antibody competition for the total anti-HBc test. The results were interpreted by comparing the specimen absorbance values (optical density, OD) at 450 nm to the cut-off (CO) value according to the manufacturer’s recommendations.

### 2.3. Reactivity to HBsAg Marker Analysis

Statistical analyses of the HBsAg reactivity of the serum samples based on the OD/CO ratio among different animal species and/or order were performed in accordance with the non-parametric Kruskal–Wallis test (95% confidence intervals). A *p*-value < 0.05 was considered to be statistically significant.

### 2.4. Molecular Tests

Viral DNA was extracted from all 487 serum samples using a DNA Purification Kit (QIAamp DNA Mini Kit, Qiagen^®^, Hilden, Germany) according to the manufacturer’s recommendations. Extracted DNA was concentrated to a final volume of 25 µL and analyzed by performing quantitative and qualitative polymerase chain reaction (PCR). To prevent cross-contamination, HBV-positive controls were not handled with animal samples.

### 2.5. Detection and Characterization of Hepadnavirus DNA

To detect hepadnavirus DNA, the samples were evaluated in duplicate by performing real-time PCR using the TaqMan^®^ method (Life Technologies^®^, Applied Biosystems, Foster City, CA, USA). The assay was performed for the pre-S2/S region as previously established [33] using the following primer pair and fluorescent probe described in the literature: forward primer (5′-GAATCCTCACAATACCGCAGAGT-3′), reverse primer (5′-GCCAAGACACACGGGTGAT-3′), and probe (5′-FAM-AAGTCCACCACGAGTCTAG-NFQ/MGB-3′) (Life Technologies^®^, Applied Biosystems, Foster City, CA, USA) [32]. Amplification was analyzed using the Applied Biosystems 7500 software. The detection limit of the assay was 5 × 10^1^ copies/μL. For this method, all samples that crossed the threshold line below 42 cycles and exhibited a characteristic sigmoid curve were considered positive.

In parallel, qualitative amplification strategies for different regions of the genome were implemented. Initially, a semi-nested PCR (PS1-S2 and PS1-SR) specific for the pre-S/S gene of HBV (~1100 bp) was performed as previously described for the first round of amplification [34] and for the second round [35]. Next, a single-round PCR specific for the core gene of HBV (430 bp) was also performed as previously described [3]. Amplicons were analyzed by electrophoresis in a 1.5% agarose gel, stained with ethidium bromide (0.2 μg/mL), and visualized under UV light. Direct sequencing of amplicons was performed to identify the amplified sequence.

### 2.6. Phylogenetic Analyses and Recombination Detection

Multiple alignments of sequences obtained from this study and GenBank were performed using MUSCLE in the SeaView v.4 program [36]. The phylogenetic relationships were estimated using (a) the maximum likelihood (ML) inference as implemented in PhyML 3 [37] under the GTR+G model of sequence evolution and (b) a Bayesian Markov chain Monte Carlo (MCMC) method as implemented in MrBayes v.3.2.5 [38]. The MCMC settings consisted of two simultaneous independent runs with four chains each that were run for 10 million generations and sampled every 100th generation, yielding 100,000 trees. After eliminating 10% of the samples as burn-in, a consensus tree was built. Statistical support of clades was measured by a heuristic search with 1000 bootstrap replicates in PhyML [39] and the Bayesian posterior probabilities in MrBayes. For the Bayesian analysis, we used a mixed nucleotide evolution model with a γ-shaped distribution of rates across sites. This model allows for the integration of selection across all best-fit models. For the ML, the best-fit evolutionary model was determined using MEGA version 7 using the Bayesian information criterion [40]. To analyze possible recombination events, the sequence alignment was analyzed with Bootscan, which was implemented in Simplot and RDP4 [41,42]. The sequences for the Bootscan analysis were grouped according to the nominal taxa clustering in the phylogenetic tree for each sequence, and the sequence of this study consisted of the query group.

## 3. Results

### 3.1. Serological Tests

**Figure 2:**
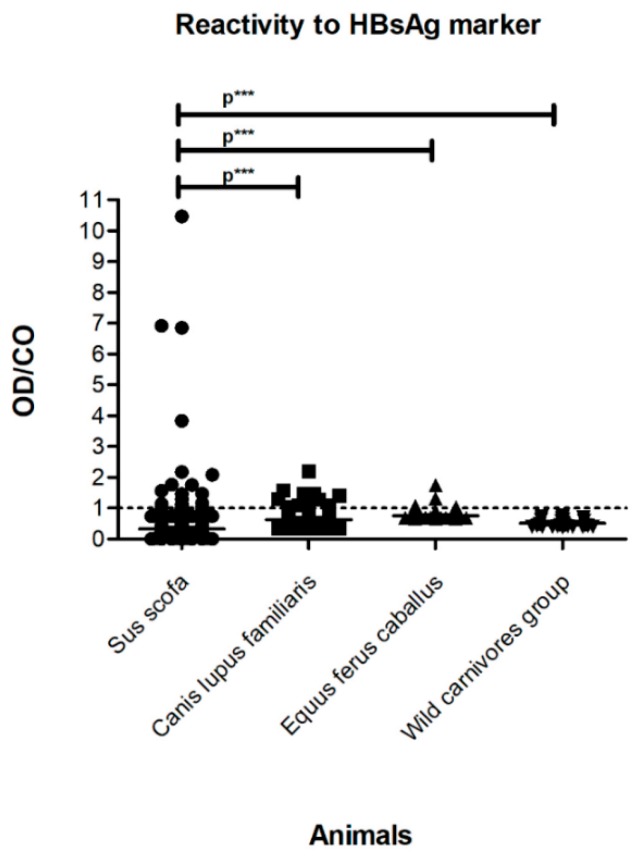
Hepatitis B virus surface antigen (HBsAg) reactivity of the serum samples in domestic and wild animals from Brazil and Uruguay. The dotted line corresponds to the cut-off line of the analysis, with reactive samples for the HBsAg marker shown above the dotted line and non-reactive samples for the same marker shown below the dotted line. A statistical analysis between groups was conducted in accordance with the Kruskal–Wallis test (95% confidence intervals). The *Sus scrofa* group corresponds to domestic pigs, wild boars, and wild pigs. The wild carnivores group corresponds to jaguar (*Panthera onca*), crab-eating foxes (*Cerdocyon thous*), maned wolves (*Chrysocyon brachyurus*), crab-eating raccoons (*Procyon cancrivorus*), and hoary foxes *(Lycalopex vetulus)*. OD/CO = absorbance value (optical density)/cut-off ratio. p*** corresponds to *p* values less than 0.001.

Based on our serological tests, 6.78% (33/487) of the samples from the different mammalian orders and biomes investigated were reactive for HBsAg, indicating the existence of an active infection. Of these, 18.18% (6/33) of the samples belonged to livestock animals (Group A), including two swine *Sus scrofa* and four horses *Equus ferus caballus*; 33.34% (11/33) corresponded to domestic dogs *Canis lupus familiaris*, which were raised wildly or free (Group B); and 48.48% (16/33) corresponded to free-roaming exotic and wild animals (Group C), including 15 wild pigs *Sus scrofa* and one jaguar *Panthera onca* (Table 1). In addition, the statistical analysis of HBsAg reactivity among different animals studied grouped by species and/or order revealed a greater reactivity index in *Sus scrofa* than the other analyzed species (*p* < 0.0001) (Figure 2).

Total anti-HBc was detected in 9.45% (46/487) of the serum samples, indicating previous exposure of these animals to the virus and viral circulation in both domestic and wild environments. Of these, 41.30% (19/46) of the samples belonged to livestock animals of Group A, including 15 swine *Sus scrofa* and 4 horses *Equus ferus caballus*; 41.30% (19/46) corresponded to 19 domestic dogs *Canis lupus familiaris* of Group B, which were raised under wild or free conditions; and 17.39% (8/46) belonged to free-roaming exotic and wild animals of Group C, including six wild pigs *Sus scrofa*, one maned wolf *Chrysocyon brachyurus*, and one crab-eating raccoon *Procyon cancrivorus*. Among the serum samples classified as reactive for total anti-HBc, 32.61% (15/46) of the samples were also reactive for HBsAg.

### 3.2. Molecular Tests

Hepadnavirus DNA was detected in 6.57% (32/487) of the investigated samples (Table 1 and Table S2), with 12.5% (4/32) in Group A, 59.38% (19/32) in Group B, and 28.13% (9/32) in Group C. Positive samples included one domestic pig *Sus scrofa* and three horses *Equus ferus caballus* (Group A); 19 domestic dogs *Canis lupus familiaris* (Group B); and seven wild pigs *Sus scrofa* and two crab-eating foxes *Cerdocyon thous* (Group C). In these samples, a domestic dog *Canis lupus familiaris* from the municipality of Cumari/Brazil was positive for hepadnavirus DNA, and it was sequenced, confirming a positive result for the pre-S/S gene of HBV (~1100 bp) (GenBank:MF991935). Molecular data concerning domestic and wildlife species from Groups A, B, and C provide evidence supporting the hepadnavirus circulation in both environments. A comparison of these environments showed that the viral DNA detection rate in *Sus scrofa* pigs was 7-fold higher in wild animals than in livestock. However, a comparison of different species from the Canidae family belonging to both environments revealed a similar molecular detection rate between wild species (*Cerdocyon thous*) and domestic animals (*Canis lupus familiaris*). The mobility of domestic dogs between both environments and their contact with other wild animals may be responsible for these similar rates between representatives of the Canidae family.

### 3.3. Phylogenetic Tree and Recombination

**Figure 3:**
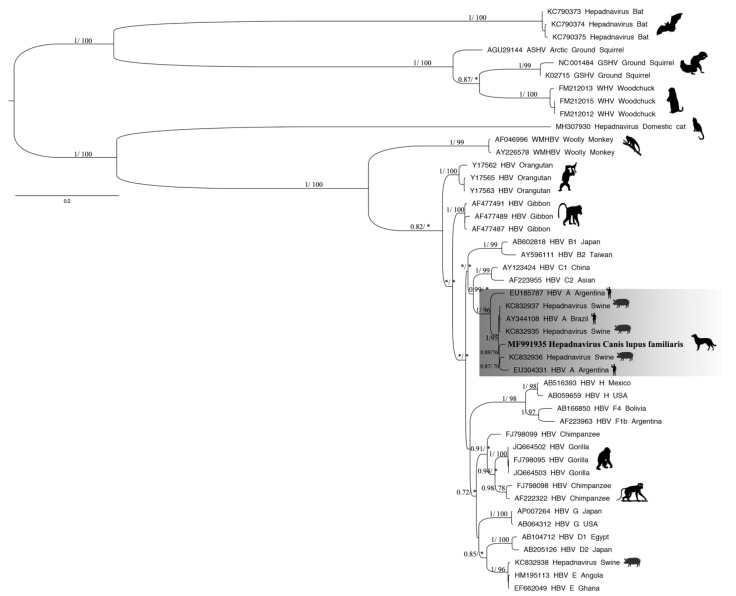
Phylogenetic relations based on the fragment of the pre-S/S gene of hepadnaviruses (1125 nt) using maximum likelihood (ML) and Bayesian methods. For each sequence used, the GenBank accession number and host are shown. The tree was rooted at the midpoint. Numbers (≥0.7/≥70) above the branches indicate node probabilities or bootstrap values (MrBayes/ML). Asterisks indicate values below 0.7/70. The sequences of this study are highlighted in bold. The scale bar indicates the evolutionary distance.

The phylogenetic inference based on the fragment of the pre-S/S gene of HBV found in representatives of the *Orthohepadnavirus* genus showed that the sequence obtained in this study formed a monophyletic clade with sequences recovered from humans and swine (branch support = 1/96) (Figure 3). The newly described sequence found in the serum from a domestic dog was highly related to sequences found in the bile of a domestic pig *Sus scrofa* from Brazil (KC832936) and the serum of an HBV patient classified as genotype A from Argentina (EU304331), with both samples collected in 2008 (0.89/76) (Figure 3). The nucleotide identity calculated between human and animal hepadnavirus strains indicated that the newly described hepadnavirus sequence found in domestic dog *Canis lupus familiaris* shared 90.4%–98.4% identity with the virus detected in swine *Sus scrofa* and 84.8%–98.2% identity with human HBV (Table S1). The Pre-S1 region of the sequence obtained in the present study compared to the closest phylogenetic sequences showed two substitutions: amino acids 38 (serine → threonine) and 94 (proline → threonine). Moreover, the S region presented one substitution in residue 173 (threonine → proline) (Figure S1). Recombination between nucleotide sequences is a major process that influences the evolution of most viruses. In many different groups of viruses, gene recombination is an important evolutionary process that generates much of the genetic diversity. The sequences obtained in this study were evaluated using a Bootscan analysis based on the fragment of the pre-S/S gene sequence, although signs of recombination were not observed. These results were reinforced by the RDP4 analysis.

## 4. Discussion

The origin of HBV is controversial [25], and many hypotheses have been postulated regarding the emergence of the virus based on its viral evolution rate and hepadnavirus nucleotide sequences from hosts already described in the literature. Human and animal relationships are likely to continue to intensify worldwide over the next several decades due in part to animal husbandry practices, the growth of the companion animal market, climate change, ecosystem disruption, the anthropogenic development of habitats, and global travel and commerce [31,43]. For example, domestic dogs and cats are the species most closely associated with humans; thus, they are the most abundant and widespread mammals in the world. Domestic dogs are present both in urban areas and altered/disturbed environments (agricultural and rural areas). As the geographical boundaries between humans and animals decrease, the possibility of pathogen spread increases. However, scientific reports seldom mention human contributions to the variety of emerging diseases that impact animals [31,44].

After serological screening, the percentages of reactive serum samples to the total anti-HBc marker in each group revealed previous viral contact between livestock (Group A) and free-roaming animal populations (Groups B and C), whether domestic or not. Regarding livestock, these data already constitute the first record of HBV-like circulation in swine herds from Uruguay and in equines from Brazil. Data in the literature have demonstrated serological and/or molecular evidence of HBV-like virus circulation in swine herds from China [19] and Brazil [20], and in chicken flocks in China [21]. Additionally, other hepatitis viruses have been demonstrated in domestic animal populations raised under confined conditions, and they include hepatitis E virus (HEV) in swine herds [45] and hepatitis C-like virus in Brazilian equines [46]. Focusing on the analyses for *Sus scrofa*, the presence of the total anti-HBc marker in the wild pig population provides evidence of the natural circulation of an HBV-like virus in free-roaming animals, regardless of their close contact with humans. These data are relevant since such circulation had already been demonstrated in swine from the domestic environment [19,20,32].

The viral circulation results in wild environments corroborate those obtained for other wild animals, such as the maned wolf and crab-eating raccoon, as well as for domestic animals that have free access to environments in which wild animals circulate, such as domestic dogs. Compared with domestic animals, dogs from the Pantanal are used for cattle work and employed in the handling and hunting of wild pigs, whereby they are exposed to the blood and fed the viscera and meat of freshly deceased animals. In the Cerrado, however, domestic dogs are exposed to a greater diversity of wild animals because they are used to hunt these animals, although such exposure occurs less frequently compared with that in the Pantanal. Domestic dogs from the Cerrado biome have a greater capacity for circulation (especially at night, when they catch wild animals), which increases the likelihood of contact with blood, saliva, and feces from these animals. Anthropogenic changes to ecosystems increase the area of shared habitats between humans and animals and thus expose both to new pathogens. Thus, given their feeding ecology and behaviors, these animals are at risk for infection through hunting and predation. Our data presented here are consistent with records of mammals and avian species harboring HBV and HBV-like viruses [11,47]. Another interesting fact is that predation can increase or decrease the prevalence of infectious diseases, depending on how it affects the frequency of infected individuals or high-quality hosts in the population [48,49]. Predation intensity on reservoir populations can alter host–pathogen dynamics [50,51] and even affect pathogen persistence in the reservoir population [44,52].

In this study, the HBsAg prevalence in livestock was 2.67% for swine and 3.20% for equines. Compared to the available data in the literature, low infection rates in livestock were also noted for swine herds from the southern and southeastern regions of Brazil (0.8%) [20], which are areas of low population endemicity for HBV. However, in highly endemic regions for HBV such as China, the overall prevalence of HBsAg in swine herds is higher (24.8%) [19]. In this study, the active infection rate between the analyzed groups was compared, and the results showed that the HBsAg detection rate was approximately 5-fold higher in free-roaming exotic and wild mammals than in confined or semi-extensive domestic animals. Distinct proportions between Groups A and C must be further investigated since they could indicate the existence of a natural source of infection. The simultaneous reactivity results for both markers (total anti-HBc and HBsAg) revealed active infection in some animals at the time of collection. However, reactive HBsAg animals were not followed up; thus, it was not possible to confirm whether the infection was acute or chronic. Furthermore, because the available sample volumes were limited, it was not possible to assess the anti-HBs levels in eventual cases of immunity after a probable resolved infection.

Cases of reactivity to HBsAg and non-reactivity to total anti-HBc may be indicative of recent ongoing infection. In this case, antibody titers against the HBV core antigen (anti-HBc) were not sufficiently high to yield a positive test [53]. Conversely, non-reactivity to HBsAg and reactivity to total anti-HBc reactive may indicate a more advanced infection because HBsAg decreases and even disappears as an acute infection resolves, and it could also indicate immunity due to natural infection and low-level chronic infection [53,54]. The analysis of HBV-like infection among different animal species and/or orders indicated greater HBsAg reactivity in *Sus scrofa* than in the other animal species (*p* < 0.0001). Because of the diversity of the investigated species, these results indicate that swine could serve as a potential experimental model of animal infection, which is important because the development of such a model represents a current challenge to advancing research on HBV replication and new antivirals [20].

This study employed a direct ELISA assay capable of detecting the HBsAg surface antigen as well as many natural and recombinant mutants with simple or multiple-point mutations. Although false-negative results are possible for hepadnaviruses other than HBV, we would like to highlight that our research is aimed at reverse zoonosis events from which human HBV-like viruses could easily circulate and eventually adapt among different niches/hosts/species. Regarding the total anti-HBc, we recognize that a perfect methodology in terms of sensitivity and specificity is not available. Every diagnostic methodology presents its limitations. However, Houareau and collaborators performed an elegant study from 2006 to 2015 with over 30 million people, and based on this significant number of samples, they demonstrated that attempting to confirm inconclusive anti-HBc results using up to three different anti-HBc methodologies was not effective [55].

The sigmoid curves of the qPCR from positive samples crossed the threshold line between 37 and 41 amplification cycles, suggesting a low viral load. This low viral load may explain the difficulty in performing partial nucleotide sequencing of the genome to confirm HBV-like virus infection, even after the adoption of DNA concentration strategies. The sensitivity and specificity of molecular diagnosis in this study were consistent with data reported previously in the literature [32]. Although qualitative PCR for ORF S and ORF C did not confirm active infection in all seroreactive animals, numerous empty spherical and filamentous particles released during the HBV replicative cycle may have been responsible for the serological diagnosis of reactive HBsAg. Furthermore, the HBsAg levels decrease more slowly than HBV-DNA levels because HBsAg has a longer half-life in serum [53]. 

The genetic distance data indicated that HBV-like canine sequences are closely related to human HBV sequences, which might suggest the likely direction of infection among different hosts. To ensure the quality of our results and exclude the possibility of contamination with viral sequences from other sources, all good laboratory practices were adopted. In addition to the use of filter tip pipettes, we used a Class II Type B2 Biological Safety Cabinet, disposable masks, coats, gloves, and other basic biosafety items. It should be reiterated that we did not manipulate the human HBV positive controls. Our results were not associated with possible contamination, and the sequence obtained in this study was genetically different from the others found in GenBank. Amino acid substitutions were found in the viral sequence obtained here, with two found in the pre-S1 region and one in the S region. The preS domain (preS1 + preS2 + S) of HBV codes for the proteins in the HBsAg compound and plays a key role in viral infection because it attaches to hepatocytes via interactions with their receptors and contains several immunogenic epitopes [56]. Authors have suggested that the preS domain determines the range of the host and specificity of hepadnaviruses. This host specificity for hepadnaviruses is likely determined by an initial infection step involving the adaptation of the N-terminal preS domain of the L protein to an unknown cellular factor. Furthermore, additional viral and host determinants may occur and should be identified [57,58,59]. Taken together, we conclude that further studies are required to clarify whether the amino acid substitutions found among hepadnaviruses imply different affinities for human and animal hepatocytes. These possibilities should be analyzed in new samplings, and could reveal whether viral circulation reflects an eventual adaption to different niches/hosts/species. Based on these data and the flexibility of circulation between different environments, domestic dogs may serve as a domestic–wild link that contributes to the introduction or permanence of the virus in the wild environment due to their ecology and behavior, which could justify the higher rates of active infection and percentages of molecular detection compared to domestic environments in this study [43,60].

## 5. Conclusions

Hepadnaviruses are zoonotic agents that circulate in different host species—both domestic and wild. Epidemiological studies focused on zoonotic agents in free-roaming animals must be performed. The presence of these infectious agents in new free-roaming species indicates the complexity of the transmission cycle due to the involvement of multiple host species; thus, molecular studies of the *Hepadnaviridae* family are warranted. However, scientific research seldom mentions the human contributions to the variety of diseases that impact animals. Future investigations of reverse zoonoses should consider both transmission routes and disease prevalence. Prospective research should also include a wider variety of etiological agents and animal species associated with HBV-like viruses. The serological and molecular results of this study suggest that the scientific literature must detail the presence and transmission of human diseases in animals (animal to animal, human to human, animal to human, and human to animal) to obtain greater knowledge and understanding of reverse zoonoses for an effective One Health response.

## Figures and Tables

**Table 1:** Prevalence of serological and molecular markers of hepadnavirus in domestic and wild animals from different sites in Brazil and Uruguay. HBc: HBV core antigen.

Groups	Common Name	Species	Country	Municipality	Total Specimens n (%)	Reactive Total Anti-HBc n (%)	Reactive HBsAg n (%)	Detected Hepadnavirus-DNA n (%)
**A—**Domestic raised in semi-extensive farms or confined	Domestic pig	*Sus scrofa*	Uruguay	Canelones/Lavalleja/Colonia	75 (37.50)	15 (20.00)	2 (2.67)	1 (1.34)
	Horse	*Equus ferus caballus*	Brazil	Seropédica	125 (62.50)	4 (3.20)	4 (3.20)	3 (2.40)
Total					200 (100)	19 (9.50)	6 (3.00)	4 (2.00)
**B—**Domestic raised wildly or free	Domestic dog	*Canis lupus familiaris*	Brazil	Corumbá/Cumari	189 (100)	19 (10.05)	11 (5.82)	19 (10.05)
Total								
**C—**Free-roaming exotic and wild mammals	Wild boar	*Sus scrofa*	Uruguay	Maldonado	11 (11.22)	0	0	0
	Wild pig	*Sus scrofa*	Brazil	Barão de Melgaço/Corumbá	61 (62.24)	6 (9.84)	15 (24.59)	7 (11.48)
	Jaguar	*Panthera onca*	Brazil	Barão de Melgaço	1 (1.02)	0	1 (100)	0
	Crab-eating fox	*Cerdocyon thous*	Brazil	Cumari	19 (19.39)	0	0	2 (10.53)
	Maned wolf	*Chrysocyon brachyurus*	Brazil	Cumari	4 (4.08)	1 (25.00)	0	0
	Crab-eating raccoon	*Procyon cancrivorus*	Brazil	Cumari	1 (1.02)	1 (100)	0	0
	Hoary fox	*Lycalopex vetulus*	Brazil	Cumari	1 (1.02)	0	0	0
Total					98 (100)	8 (8.16)	16 (16.33)	9 (9.18)
				**Total**	**487 (100)**	**46 (9.45)**	**33 (6.78)**	**32 (6.57)**

## Supplementary Materials

The following are available online at https://www.mdpi.com/1660-4601/16/15/2679/s1: **Table S1**: Nucleotide identity between hepadnavirus strains from domestic dogs and other species. Legend: The nucleotide identity matrix was constructed by using the partial nucleotide sequence of pre-S/S (1125 nt) from HBV and related viruses. For each sequence used, the GenBank accession number and species infected are shown. **Table S2**: Serological results of animals positive for hepadnavirus DNA (PCR). **Figure S1**: Schematic representation of the pre-S/S gene of HBV. The sequence shows three changes in the amino acids.

## References

[1] Tiollais P., Pourcel C., Dejean A. (1985). The hepatitis B virus. Nature.

[2] Mandart E., Kay A., Galibert F. (1984). Nucleotide sequence of a cloned duck hepatitis B virus genome: Comparison with woodchuck and human hepatitis B virus sequences. J. Virol..

[3] Lanford R.E., Chavez D., Brasky K.M., Burns R.B., Rico-Hesse R. (1998). Isolation of a hepadnavirus from the woolly monkey, a New World primate. Proc. Natl. Acad. Sci. USA.

[4] Warren K.S., Heeney J.L., Swan R.A., Heriyanto, Verschoor E.J. (1999). A new group of hepadnaviruses naturally infecting orangutans (*Pongo pygmaeus*). J. Virol..

[5] Grethe S., Heckel J.O., Rietschel W., Hufert F.T. (2000). Molecular epidemiology of hepatitis B virus variants in nonhuman primates. J. Virol..

[6] Hu X., Margolis H.S., Purcell R.H., Ebert J., Robertson B.H. (2000). Identification of hepatitis B virus indigenous to chimpanzees. Proc. Natl. Acad. Sci. USA.

[7] MacDonald D.M., Holmes E.C., Lewis J.C., Simmonds P. (2000). Detection of hepatitis B virus infection in wild-born chimpanzees (*Pan troglodytes verus*): Phylogenetic relationships with human and other primate genotypes. J. Virol..

[8] Summers J., Smolec J.M., Snyder R. (1978). A virus similar to human hepatitis B virus associated with hepatitis and hepatoma in woodchucks. Proc. Natl. Acad. Sci. USA.

[9] Marion P.L., Oshiro L.S., Regnery D.C., Scullard G.H., Robinson W.S. (1980). A virus in Beechey ground squirrels that is related to hepatitis B virus of humans. Proc. Natl. Acad. Sci. USA.

[10] Trueba D., Phelan M., Nelson J., Beck F., Pecha B.S., Brown R.J., Varmus H.E., Ganem D. (1985). Transmission of ground squirrel hepatitis virus to homologous and heterologous hosts. Hepatology.

[11] Drexler J.F., Geipel A., Konig A., Corman V.M., van Riel D., Leijten L.M., Bremer C.M., Rasche A., Cottontail V.M., Maganga G.D. (2013). Bats carry pathogenic hepadnaviruses antigenically related to hepatitis B virus and capable of infecting human hepatocytes. Proc. Natl. Acad. Sci. USA.

[12] Mason W.S., Aldrich C., Summers J., Taylor J.M. (1982). Asymmetric replication of duck hepatitis B virus DNA in liver cells: Free minus-strand DNA. Proc. Natl. Acad. Sci. USA.

[13] Jilbert A.R.R., Freiman J.S.S., Gowans E.J.J., Holmes M., Cossart Y.E.E., Burrell C.J.J. (1987). Duck hepatitis B virus DNA in liver, spleen, and pancreas: Analysis by in situ and southern blot hybridization. Virology.

[14] Sprengel R., Kaleta E.F., Will H. (1988). Isolation and characterization of a hepatitis B virus endemic in herons. J. Virol..

[15] Chang S.F., Netter H.J., Bruns M., Schneider R., Frölich K., Will H. (1999). A new avian hepadnavirus infecting snow geese (*Anser caerulescens*) produces a significant fraction of virions containing single-stranded DNA. Virology.

[16] Pult I., Netter H.J., Bruns M., Prassolov A., Sirma H., Hohenberg H., Chang S.F., Frölich K., Krone O., Kaleta E.F. (2001). Identification and analysis of a new hepadnavirus in white storks. Virology.

[17] Triyatni M., Ey P., Tran T., Le Mire M., Qiao M., Burrell C., Jilbert A. (2001). Sequence comparison of an Australian duck hepatitis B virus strain with other avian hepadnaviruses. J. Gen. Virol..

[18] Guo H., Mason W.S., Aldrich C.E., Saputelli J.R., Miller D.S., Jilbert A.R., Newbold J.E. (2005). Identification and characterization of avihepadnaviruses isolated from exotic anseriformes maintained in captivity. J. Virol..

[19] Li W., She R., Liu L., You H., Yin J. (2010). Prevalence of a virus similar to human hepatitis B virus in swine. Virol. J..

[20] Vieira Y.R., Silva M.F., Santos D.R., Vieira A.A., Ciacci-Zanella J.R., Barquero G., do Lago B.V., Gomes S.A., Pinto M.A., De Paula V.S. (2015). Serological and molecular evidence of hepadnavirus infection in swine. Ann. Agric. Environ. Med. Ann. Agric. Env. Med..

[21] Tian J., Xia K., She R., Li W., Ding Y., Wang J., Chen M., Yin J. (2012). Detection of hepatitis B virus in serum and liver of chickens. Virol. J..

[22] Suh A., Weber C.C., Kehlmaier C., Braun E.L., Green R.E., Fritz U., Ray D.A., Ellegren H. (2014). Early Mesozoic coexistence of Amniotes and hepadnaviridae. PLoS Genet..

[23] Gilbert C., Meik J.M., Dashevsky D., Card D.C., Castoe T.A., Schaack S. (2014). Endogenous hepadnaviruses, bornaviruses and circoviruses in snakes. Proc. Biol. Sci..

[24] Hahn C.M., Iwanowicz L.R., Cornman R.S., Conway C.M., Winton J.R., Blazer V.S. (2015). Characterization of a novel hepadnavirus in the White Sucker (*Catostomus commersonii*) from the great lakes region of the United States. J. Virol..

[25] Paraskevis D., Angelis K., Magiorkinis G., Kostaki E., Ho S.Y.W., Hatzakis A. (2015). Dating the origin of hepatitis B virus reveals higher substitution rate and adaptation on the branch leading to F/H genotypes. Mol. Phylogenet. Evol..

[26] Komatsu H. (2014). Hepatitis B virus: Where do we stand and what is the next step for eradication?. World J. Gastroenterol..

[27] World Health Organization. http://www.who.int/topics/hepatitis/en/.

[28] Lozano R., Naghavi M., Foreman K., Lim S., Shibuya K., Aboyans V., Abraham J., Adair T., Aggarwal R., Ahn S.Y. (2012). Global and regional mortality from 235 causes of death for 20 age groups in 1990 and 2010: A systematic analysis for the Global Burden of Disease Study 2010. Lancet.

[29] Makuwa M., Souquière S., Clifford S.L., Mouinga-Ondeme A., Bawe-Johnson M., Wickings E.J., Latour S., Simon F., Roques P. (2005). Identification of hepatitis B virus genome in faecal sample from wild living chimpanzee (*Pan troglodytes troglodytes*) in Gabon. J. Clin. Virol..

[30] Bonvicino C.R., Moreira M.A., Soares M.A. (2014). Hepatitis B virus lineages in mammalian hosts: Potential for bidirectional cross-species transmission. World J. Gastroenterol..

[31] Messenger A.M., Barnes A.N., Gray G.C. (2014). Reverse zoonotic disease transmission (Zooanthroponosis): A systematic review of seldom-documented human biological threats to animals. PLoS ONE.

[32] Vieira Y.R., dos Santos D.R.L., Portilho M.M., Velloso C.E.P., Arissawa M., Villar L.M., Pinto M.A., de Paula V.S. (2014). Hepadnavirus detected in bile and liver samples from domestic pigs of commercial abattoirs. BMC Microbiol..

[33] Portilho M.M. (2013). Desenvolvimento de Testes de Detecção e Quantificação do Vírus da Hepatite B em Amostras de Soro e Fluido Oral.

[34] Niel C., Moraes M.T., Gaspar A.M., Yoshida C.F., Gomes S.A. (1994). Genetic diversity of hepatitis B virus strains isolated in Rio de Janeiro, Brazil. J. Med. Virol.

[35] Motta-Castro A.R.C., Martins R.M.B., Yoshida C.F.T., Teles S.A., Paniago A.M., Lima K.M.B., Gomes S.A. (2005). Hepatitis B virus infection in isolated Afro-Brazilian communities. J. Med. Virol..

[36] Gouy M., Guindon S., Gascuel O. (2010). SeaView Version 4: A multiplatform graphical user interface for sequence alignment and phylogenetic tree building. Mol. Biol. Evol..

[37] Guindon S., Gascuel O. (2003). A simple, fast and accurate algorithm to estimate large phylogenies by maximum-likelihood. Syst. Biol..

[38] Ronquist F., Teslenko M., van Der Mark P., Ayres D.L., Darling A., Höhna S., Larget B., Liu L., Suchard M.A., Huelsenbeck J.P. (2012). Mrbayes 3.2: Efficient bayesian phylogenetic inference and model choice across a large model space. Syst. Biol..

[39] Anisimova M., Gascuel O., Sullivan J. (2006). Approximate likelihood-ratio test for branches: A fast, accurate, and powerful alternative. Syst. Biol..

[40] Kumar S., Stecher G., Tamura K. (2016). MEGA7: Molecular evolutionary genetics analysis version 7.0 for bigger datasets. Mol. Biol. Evol..

[41] Lole K.S., Bollinger R.C., Paranjape R.S., Gadkari D., Kulkarni S.S., Novak N.G., Ingersoll R., Sheppard H.W., Ray S.C. (1999). Full-length human immunodeficiency virus type 1 genomes from subtype C-infected seroconverters in India, with evidence of intersubtype recombination. J. Virol..

[42] Martin D.P., Murrell B., Golden M., Khoosal A., Muhire B. (2015). RDP4: Detection and analysis of recombination patterns in virus genomes. Virus Evol..

[43] Hubálek Z. (2003). Emerging human infectious diseases: Anthroponoses, zoonoses, and sapronoses. Emerg. Infect. Dis..

[44] Guterres A., de Lemos E.R.S. (2018). Hantaviruses and a neglected environmental determinant. One Heal..

[45] Dos Santos D.R.L., Vitral C.L., de Paula V.S., Marchevsky R.S., Lopes J.F., Gaspar A.M.C., Saddi T.M., Júnior N.C.D.M., Guimarães F.D.R., Júnior J.G.C. (2009). Serological and molecular evidence of hepatitis E virus in swine in Brazil. Vet. J..

[46] Figueiredo A.S., Lampe E., do Espírito-Santo M.P., do Amaral Mello F.C., de Almeida F.Q., de Lemos E.R.S., Godoi T.L.O.S., Dimache L.A.G., dos Santos D.R.L., Villar L.M. (2015). Identification of two phylogenetic lineages of equine hepacivirus and high prevalence in Brazil. Vet. J..

[47] Yang J., Xi Q., Deng R., Wang J., Hou J., Wang X. (2007). Identification of interspecies recombination among hepadnaviruses infecting cross-species hosts. J. Med. Virol..

[48] Borer E.T., Mitchell C.E., Power A.G., Seabloom E.W. (2009). Consumers indirectly increase infection risk in grassland food webs. Proc. Natl. Acad. Sci. USA.

[49] Holt R.D., Roy M. (2007). Predation can increase the prevalence of infectious disease. Am. Nat..

[50] Dwyer G., Dushoff J., Yee S.H. (2004). The combined effects of pathogens and predators on insect outbreaks. Nature.

[51] Packer C., Holt R.D., Hudson P.J., Lafferty K.D., Dobson A.P. (2003). Keeping the herds healthy and alert: Implications of predator control for infectious disease. Ecol. Lett..

[52] Hall S.R., Duffy M.A., Cáceres C.E. (2005). Selective predation and productivity jointly drive complex behavior in host-parasite systems. Am. Nat..

[53] Gerlich W.H. (2013). Medical virology of hepatitis B: How it began and where we are now. Virol. J..

[54] Centers for Disease Control and Prevention. http://www.cdc.gov/hepatitis/HBV/index.htm.

[55] Houareau C., Offergeld R. (2019). Anti-HBc screening— Is it worth the effort? Results of a 10-year surveillance programme covering more than 30 million donations in Germany. Vox Sang..

[56] Shouval D., Roggendorf H., Roggendorf M. (2015). Enhanced immune response to hepatitis B vaccination through immunization with a Pre-S1/Pre-S2/S vaccine. Med. Microbiol. Immunol..

[57] Ishikawa T., Ganem D. (1995). The pre-S domain of the large viral envelope protein determines host range in avian hepatitis B viruses. Proc. Natl. Acad. Sci. USA.

[58] Chouteau P., Le Seyec J., Cannie I., Nassal M., Guguen-Guillouzo C., Gripon P. (2001). A short N-proximal region in the large envelope protein harbors a determinant that contributes to the species specificity of human hepatitis B virus. J. Virol..

[59] Glebe D., Urban S. (2007). Viral and cellular determinants involved in hepadnaviral entry. World J. Gastroenterol..

[60] Feagins A.R., Opriessnig T., Huang Y.W., Halbur P.G., Meng X.J. (2008). Cross-species infection of specific-pathogen-free pigs by a genotype 4 strain of human hepatitis E virus. J. Med. Virol..

