# Supplementary material for: Evaluation of HBV-Like Circulation in Wild and Farm Animals from Brazil and Uruguay
Source: Int J Environ Res Public Health. 2019 Jul 26;16(15):2679. doi: 10.3390/ijerph16152679 (PMC6695864; doi:10.3390/ijerph16152679)
Supplement: Supplementary file 1 [file ijerph-16-02679-s001.pdf]

Table S1: Nucleotide identity between hepadnavirus strains from domestic dogs and other species. Legend: The nucleotide identity matrix was constructed using a partial nucleotide sequence of pre-S/S (1125 nt) of HBV and related viruses. For each sequence used, the GenBank accession number and infected species are shown.

GenBank	Host	Country	Nucleotide identity
KC832937	Swine	Brazil	98.4%
KC832935			98.3%
KC832936			97.8%
KC832938			90.4%
AY344108	Human (HBV A)	Brazil	98.2%
EU304331	Human (HBV A)	Argentina	97.5%
EU185787	Human (HBV A)	Argentina	95.6%
AP007264	Human (HBV G)	Japan	92.3%
AF223955	Human (HBV C2)	Asian	92.1%
AY123424	Human (HBV C1)	China	92.1%
AB064312	Human (HBV G)	USA	92.1%
EF662049	Human (HBV E)	Ghana	90.4%
HM195113	Human (HBV E)	Angola	90.3%
AB602818	Human (HBV B1)	Japan	90.3%
AB104712	Human (HBV D1)	Egypt	89.9%
AB205126	Human (HBV D2)	Japan	89.5%
AY596111	Human (HBV B2)	Taiwan	89.2%
AB516393	Human (HBV H)	Mexico	87.0%
AB059659	Human (HBV H)	USA	86.7%
AF223963	Human (HBV F1b)	Argentina	85.1%
AB166850	Human (HBV F4)	Bolivia	84.8%
JQ664502	Gorilla	Cameroon	91.1%
FJ798095			90.9%
JQ664503			
Y17563	Orangutan	Indonesia	90.7%
Y17565			90.7%
Y17562			90.3%
FJ798099	Chimpanzee	Cameroon	90.5%
FJ798098			90.3%
AF222322		London	90.3%
AF477487	Gibbon	Thailand	90.4%
AF477491			
AF477489			89.9%
AF046996	Woolly Monkey	EUA	76.2%
AY226578			76.1%
MH307930	Domestic cat	Australia	64.4%
KC790375	Bat	Gabon	57.3%
KC790374			57.3%
KC790373			57.1%
U29144	Ground Squirrel	Alaska	51.2%
NC 001484		USA	50.8%
K02715			50.8%
FM212013	Woodchuck	EUA	50.8%
FM212015			50.5%

Table S2. Serological results of positive animals for hepadnavirus DNA (PCR).

Groups	Species	N°	Serological result	
			Anti-HBc total	HBsAg
A	*Sus scrofa*	SM124	-	-
	Equus ferus caballus	HR26	+	-
		HR91	-	-
		HR95	-	-
B	*Canis lupus familiaris*	CN06	-	-
		CN13	-	-
		CN17	-	-
		CN60	-	-
		CN67	-	-
		CN70	-	-
		CN79	-	-
		CN83	-	-
		CN106	-	-
		CNOX	+	+
		CD71	-	-
		CD83	-	-
		CD127	-	-
		CD131	+	-
		CD145	-	-
		**CD156***	-	-
		CD161	+	-
		CD179	+	-
		CD187	-	-
C	*Sus scrofa*	SSRS302	-	-
		SSRS306	-	-
		5812	-	-
		5814	-	-
		5820	-	+
		5841	-	-
		5842	-	-
	Cerdocyon thous	PCMC919	-	-
		BcTh941	-	-

* Sequenced sample.

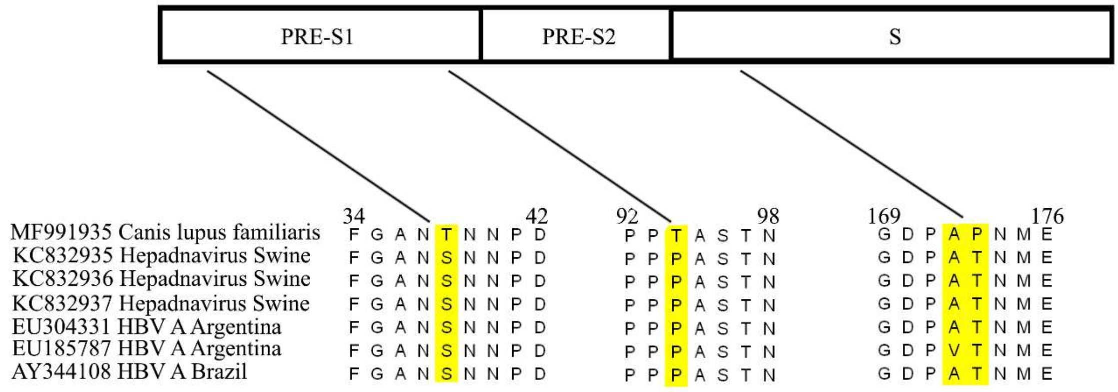

Figure S1. Schematic representation of the pre-S/S gene of HBV. The sequence shows three changes in the amino acids.
